# Trends in coverage following an equity-oriented strategy for introducing new vaccines, Peru, 2004–2022

**DOI:** 10.2471/BLT.24.292434

**Published:** 2025-02-25

**Authors:** Larissa A N Silva, Francine S Costa, Bianca O Cata-Preta, Luis Huicho, Claudio F Lanata, Maria Ana Mendoza Araujo, Theresa J Ochoa, Tewodaj Mengistu, Dan Hogan, Aluisio J D Barros, Cesar G Victora

**Affiliations:** a International Center for Equity in Health and Postgraduate Program in Epidemiology, Federal University of Pelotas, Marechal Deodoro, 1160 Pelotas, RS, 96020–220, Brazil.; b Public Health Department, Federal University of Parana, Curitiba, Brazil.; c Facultad de Medicina, Universidad Peruana Cayetano Heredia, Lima, Peru.; d Instituto de Investigación Nutricional, Lima, Peru.; e Lima, Peru.; f Instituto de Medicina Tropical Alexander von Humboldt, Universidad Peruana Cayetano Heredia, Lima, Peru.; g Gavi, the Vaccine Alliance, Geneva, Switzerland.

## Abstract

**Objective:**

To evaluate the outcome of Peru’s strategy to introduce new vaccines in the poorest regions with high child mortality rates.

**Methods:**

We analysed data from nationally representative annual health surveys conducted between 2004 and 2022. We examined associations between vaccine coverage and poverty (proportion of households in the poorest 40% of the national wealth index) at the ecological level using the country’s 25 regions and at the individual child level using household wealth quintiles. We obtained vaccination data from home-based records.

**Findings:**

The surveys included 49 023 children aged 18–29 months. In the ecological analyses, coverage for *Haemophilus influenzae* type b, pneumococcal conjugate and rotavirus vaccines was positively associated with poverty prevalence in the initial post-introduction period, but these associations disappeared over time. In contrast, the individual-level analyses indicated that children from wealthier families were consistently more likely to be vaccinated than children from poorer families. In the most recent period (2018–2022), vaccination coverage in the wealthiest quintile was about 10 percentage points higher than in the poorest quintile. Coverage levels for boys and girls were similar. Children whose low-income families were enrolled in the *Juntos* cash transfer programme had higher coverage than the rest of the population.

**Conclusion:**

The strategy increased coverage in the poorest districts initially and, as national coverage grew, regional disparities were eliminated. However, socioeconomic differences persisted, with wealthier children maintaining higher vaccination rates throughout the study. To eliminate these disparities, geographic targeting should be complemented with household-level targeting.

## Résumé

**Objectif:**

Évaluer les résultats de la stratégie péruvienne de lancement de nouveaux vaccins dans les régions les plus pauvres présentant des taux de mortalité infantile élevés.

**Méthodes:**

Nous avons analysé les données d’enquêtes de santé annuelles représentatives à l’échelle nationale, menées entre 2004 et 2022. Nous avons examiné les associations entre la couverture vaccinale et la pauvreté (proportion de ménages dans les 40% les plus pauvres de l’indice de richesse nationale) au niveau écologique en utilisant les 25 régions du pays et au niveau de l’enfant individuel en utilisant les quintiles de richesse des ménages. Les données sur les vaccinations ont été obtenues à partir des registres de vaccination à domicile.

**Résultats:**

Les enquêtes ont porté sur 49 023 enfants âgés de 18 à 29 mois. Dans les analyses écologiques, la couverture vaccinale contre l’*Haemophilus influenzae* de type B, le pneumocoque conjugué et le rotavirus était positivement associée à la prévalence de la pauvreté dans la période suivant le lancement du vaccin, mais ces associations disparaissaient avec le temps. En revanche, les analyses au niveau individuel indiquaient que les enfants issus de familles plus aisées étaient systématiquement davantage susceptibles d’être vaccinés que les enfants issus de familles plus pauvres. Au cours de la période la plus récente (2018–2022), la couverture vaccinale dans le quintile des familles les plus aisées était supérieure d’environ 10 points de pourcentage à celle du quintile des familles les plus pauvres. Les niveaux de couverture vaccinale pour les garçons et les filles étaient similaires. Les enfants dont les familles à faible revenu étaient inscrites au programme de transfert monétaire Juntos avaient une couverture plus élevée que le reste de la population.

**Conclusion:**

Cette stratégie a dans un premier temps poussé à la hausse la couverture dans les districts les plus pauvres et, à mesure que la couverture nationale augmentait, les inégalités régionales ont disparu. Cependant, des différences socio-économiques ont persisté, les enfants de familles plus aisées conservant des taux de vaccination plus élevés tout au long de l’étude. Pour éliminer ces disparités, le ciblage géographique devrait se compléter d’un ciblage au niveau des ménages.

## Resumen

**Objetivo:**

Evaluar los resultados de la estrategia de Perú para introducir nuevas vacunas en las regiones más pobres con altas tasas de mortalidad infantil.

**Métodos:**

Se analizaron datos de encuestas nacionales de salud representativas realizadas anualmente entre 2004 y 2022. Se examinaron las asociaciones entre la cobertura de vacunación y la pobreza (porcentaje de hogares en el 40% más pobre del índice de riqueza nacional) a nivel ecológico, utilizando las 25 regiones del país, y a nivel individual, empleando los quintiles de riqueza de los hogares. Los datos de vacunación se obtuvieron de registros domiciliarios.

**Resultados:**

Las encuestas incluyeron a 49 023 niños de entre 18 y 29 meses. En los análisis ecológicos, la cobertura de las vacunas contra *Haemophilus influenzae* tipo b, neumococo conjugada y rotavirus se asoció positivamente con la prevalencia de pobreza en el período inicial posterior a la introducción; sin embargo, estas asociaciones desaparecieron con el tiempo. En contraste, los análisis a nivel individual indicaron que los niños de familias con mayor nivel de riqueza tenían una mayor probabilidad de estar vacunados en comparación con aquellos de familias más pobres. En el período más reciente (2018-2022), la cobertura de vacunación en el quintil de mayor riqueza fue aproximadamente 10 puntos porcentuales superior a la del quintil más pobre. Los niveles de cobertura fueron similares entre niños y niñas. Los niños de familias de bajos ingresos inscritas en el programa de transferencias monetarias *Juntos* presentaron una cobertura superior a la del resto de la población.

**Conclusión:**

La estrategia permitió aumentar inicialmente la cobertura en los distritos más pobres y, a medida que la cobertura nacional creció, se eliminaron las disparidades regionales. Sin embargo, persistieron las diferencias socioeconómicas, con tasas de vacunación más elevadas entre los niños de familias con mayor nivel de riqueza durante todo el período de estudio. Para eliminar estas disparidades, la focalización geográfica debe complementarse con una focalización a nivel de los hogares.

## ملخص

الغرض تقييم نتائج استراتيجية بيرو لطرح لقاحات جديدة في أفقر المناطق ذات المعدلات المرتفعة لوفيات الأطفال.

الطريقة قمنا بتحليل البيانات من المسوح الصحية السنوية الممثلة على المستوى الوطني، والتي تم إجراؤها خلال الفترة من 2004 إلى 2022. قمنا بفحص الارتباطات بين تغطية اللقاح والفقر (نسبة الأسر في أفقر %40 من مؤشر الثروة الوطنية) على المستوى البيئي باستخدام 25 منطقة في الدولة، وعلى مستوى الطفل الفردي باستخدام الشرائح المئوية للثروة الأسرية. حصلنا على بيانات التطعيم من السجلات المنزلية.

النتائج شملت المسوح 49023 طفلاً تتراوح أعمارهم بين 18 و29 شهرًا. في التحليلات البيئية، ارتبطت التغطية بلقاح المستدمية النزلية من النوع ب، واللقاح المقترن بالمكورات الرئوية، واللقاح الفيروسي العجلي، بشكل إيجابي بانتشار الفقر في فترة ما بعد التقديم الأولية، لكن هذه الارتباطات اختفت بمرور الوقت. وعلى النقيض من ذلك، أشارت التحليلات على مستوى الفرد إلى أن الأطفال من الأسر الأكثر ثراءً، كانوا أكثر احتمالية للتطعيم بشكل متسق، من الأطفال من الأسر الأكثر فقراً. في الفترة الأخيرة (2018 إلى 2022)، كانت تغطية التطعيم في الخمس الأكثر ثراءً أعلى بنحو 10 نقاط مئوية من الخمس الأكثر فقراً. كانت مستويات التغطية للبنين والبنات متشابهة. الأطفال الذين تم إدراج أسرهم ذات الدخل المنخفض في برنامج *Juntos * التحويلات النقدية، كانوا أكثر تغطية من بقية السكان.

الاستنتاج زادت الاستراتيجية من التغطية في أفقر المناطق في البداية، ومع نمو التغطية الوطنية، تم القضاء على التفاوتات الإقليمية. ومع ذلك، استمرت الاختلافات الاجتماعية والاقتصادية، حيث حافظ الأطفال الأكثر ثراءً على معدلات تطعيم أعلى طوال الدراسة. للقضاء على هذه التفاوتات، يجب استكمال الاستهداف الجغرافي بالاستهداف على مستوى الأسرة.

## 摘要

**目的:**

评估秘鲁在儿童死亡率很高的最贫困地区推行新疫苗引入战略取得的成果。

**方法:**

我们分析了 2004 年至 2022 年期间开展的全国代表性年度健康调查的数据。我们在生态层面（覆盖该国 25 个地区）和在儿童个体层面（利用家庭财富五分位数）研究了疫苗接种覆盖率与贫困之间的关联性（国家财富指数中 40% 最贫困家庭的疫苗接种覆盖率）。我们从家庭（医疗）记录中获得了疫苗接种数据。

**结果:**

调查对象包括 49,023 名 18-29 个月大的幼儿。根据生态分析，乙型流感嗜血杆菌疫苗、肺炎球菌结合疫苗和轮状病毒疫苗的接种覆盖率与引入后初期的贫困率呈正相关，但随着时间的推移，这些相关性逐渐消失。相比之下，个体层面的分析表明，富裕家庭的儿童始终比贫困家庭的儿童更有可能接种疫苗。在最近这段时间（2018-2022 年）里，与最贫穷的五分之一家庭中的儿童相比，最富有的五分之一家庭中的儿童的疫苗接种覆盖率高出约 10 个百分点。男孩和女孩的疫苗接种覆盖率水平相似。对于参与了 Juntos 现金转移方案的低收入家庭，其儿童的疫苗接种覆盖率高于同阶层其余儿童。

**结论:**

该战略最初提高了最贫困地区的疫苗接种覆盖率，而且随着全国疫苗接种覆盖率逐步升高，消除了地区差异。尽管如此，社会经济差异依然存在，在整个研究过程中，来自富裕家庭的儿童始终具有较高的疫苗接种率。为了消除这些差异，地域目标应与家庭层面的目标相辅相成。

## Резюме

**Цель:**

Оценить результаты перуанской стратегии внедрения новых вакцин в беднейших регионах страны с высоким уровнем детской смертности.

**Методы:**

Авторы проанализировали данные национальных репрезентативных ежегодных опросов о состоянии здоровья, проведенных в период с 2004 по 2022 г. Изучалась связь между охватом вакцинированием, уровнем бедности (долей семей, относящихся к беднейшему населению, то есть тех, благосостояние которых составляет не более 40% показателя национального благосостояния) на уровне окружения в 25 регионах страны, а также на уровне индивидуального здоровья детей с использованием распределения здоровья семейств по квинтилям. Данные о вакцинировании были получены на основании внутренней национальной документации.

**Результаты:**

В исследование было включено 49 023 ребенка в возрасте от 18 до 29 месяцев. На уровне окружения охват вакцинами против *Haemophilus influenzae* типа b, ротавируса и пневмококковой конъюгированной вакциной положительно ассоциировался с преобладанием бедности сразу после внедрения вакцинирования, но со временем эта связь размылась. Напротив, на индивидуальном уровне оказалось, что дети из более зажиточных семей с большей вероятностью могли быть вакцинированы, нежели дети из более бедных семей. В последние годы (2018–2022 гг.) охват вакцинированием в наиболее богатом квинтиле был почти на 10% выше, чем в беднейшем. Охват был одинаковым как для мальчиков, так и для девочек. Дети из семей с низким доходом, получавших наличные средства по программе *Juntos*, имели более высокий уровень охвата вакцинированием, нежели во всей остальной популяции.

**Вывод:**

Сначала стратегия повысила охват в беднейших районах страны, но по мере роста национального охвата различия между регионами были устранены. Однако сохранилось социально-экономическое неравенство, и на всем протяжении исследования дети из более зажиточных семей имели более высокий уровень вакцинирования. Чтобы устранить эти различия, следует применить географический подход с целенаправленными усилиями на уровне отдельных семей.

## Introduction

Many inequalities in child health status and intervention coverage have been documented in low- and middle-income countries.^1^ Childhood immunization coverage is consistently higher in urban than rural areas, and in the wealthiest regions and households of nearly all countries.^2^^–^^5^ This situation reflects the fact that new health interventions tend to be introduced first in better-off, easy-to-reach subpopulations of a country,^6^ leading to increased inequalities, at least in the short term.^7^ Such prevailing patterns have been described as the “mainstream approaches that incrementally increase coverage from the easier to the more difficult-to-reach populations.”^8^ An alternative approach is to prioritize service delivery to the poorest and most marginalized communities.^9^ Indeed, a multicountry modelling exercise confirmed that such a strategy would be more effective and cost-effective than mainstream approaches.^8^

Faced with important child health inequalities among its 25 regions and the strong association between child mortality and socioeconomic conditions,^10^^,^^11^ the mainstream logic was challenged in Peru. Accordingly, Peruvian policy-makers decided to introduce new vaccines first in the country’s most vulnerable regions, classified according to the poverty measurement system of the National Institute of Statistics and Computing. This classification relied on data from censuses and surveys on poverty, education, health and access to different services which provided geographically disaggregated data. As a result, a new vaccine against *Haemophilus influenzae* type b (Hib) in 1998 was targeted to the poorest regions of the country where child mortality was highest.^10^^,^^11^ The high price and limited availability of Hib vaccine doses and the small number of children living in these regions (mainly in the Amazon and the Andes) contributed to this decision to prioritize regions classified as extremely poor.^12^ As well as making vaccines available, the health ministry worked with the Pan American Health Organization (PAHO) and the United Nations Children’s Fund to train health workers and improve the infrastructure needed for immunization, including the cold chain.

Similar strategies were adopted when rotavirus and pneumococcal conjugate vaccines were introduced in 2008 and 2009, respectively.^13^^,^^14^ After the initial period of geographical targeting, the new vaccines, as well as the training activities and infrastructure improvements, were scaled up nationally with the aim of reaching universal coverage.^13^^,^^14^

The Peruvian strategy has been highlighted as an innovative, pro-equity and results-based approach.^10^^,^^15^ Yet no formal evaluations of this strategy are available. We therefore assessed whether the Peruvian strategy effectively resulted in higher vaccine coverage in the poorest regions than in the remainder of the country over two decades. We also investigated whether the approach led to higher coverage among children from poor and rural households compared with other children and children from families enrolled in the *Juntos* conditional cash transfer programme. This programme was launched in 2005 and started in the country’s poorest regions, which were identified through the national targeting system based on a composite poverty indicator.^16^^,^^17^ We also assessed sex differences in coverage. We focused on the introduction of the Hib, rotavirus and pneumococcal vaccines, using data from annual population-based surveys in all 25 regions in the country.

## Methods

We initially conducted the analyses at an ecological level, with each of the 25 regions in the country as a unit. Next, we undertook individual child-level analyses.

### Study samples

We used data from the annual household demographic and health surveys (*Encuesta Demográfica y de Salud Familiar*) that have been conducted in Peru by the National Institute of Statistics and Computing since 2004. The surveys are highly comparable to Demographic and Health Surveys with the samples representative at the regional level.^18^^–^^20^

### Variables

The outcome variables were vaccine coverage in children aged 18–29 months, the age group traditionally used in Peru. At this age, in accordance with the vaccine schedule adopted by the health ministry, children should have received three doses of the Hib vaccine (at ages 2, 4 and 6 months) and pneumococcal conjugate vaccine (at ages 2, 4 and 12 months), and two doses of the rotavirus vaccine (at ages 2 and 4 months).^13^ The Hib vaccine was introduced in 1998 as a standalone vaccine, but since 2007 it has been given as part of the pentavalent vaccine (diphtheria–tetanus–pertussis–Hib–hepatitis B).^13^ We included Hib vaccine coverage as standalone and the pentavalent formulations.

In the annual surveys, the interviewers recorded the number of doses from official vaccination records given to caregivers to keep at home for Hib-containing vaccines. Respondents were asked to recall the number of doses if such cards were unavailable. However, the interviewers did not ask this additional question for the pneumococcal conjugate and rotavirus vaccines. Hib vaccine coverage levels that also considered reported doses were about 7 percentage points higher than those solely derived from records (online repository).^21^ For consistency across vaccines, we opted to restrict the primary analyses to doses reported in home-based records for the three vaccines.

The ecological analyses included two explanatory variables measured in the same surveys as immunization coverage: (i) prevalence of poverty, calculated as the percentage of children in each region living in households classified in the poorest 40% of the national wealth index; and (ii) enrolment in the *Juntos* programme, calculated as the percentage of children whose families were enrolled (these data were available for 2008–2021).

Poverty was assessed through the wealth index derived through principal component analysis of variables such as ownership of durable goods (for example, televisions, bicycles and cars), as well as housing characteristics (for example, type of flooring and roofing materials, access to utilities such as electricity and clean water, and ownership of land). Households were then divided into wealth quintiles.^22^ The asset indices and wealth quintiles were calculated by the survey analytical teams and included in the downloadable data sets.

We used household wealth quintiles and *Juntos* enrolment as stratifiers in the individual-level analyses, as well as the child’s sex (as reported by the respondent) and place of residence (urban or rural, as defined by National Institute of Statistics and Computing).

### Data analyses

For the analyses we used *Stata* version 18 (StataCorp. LP, College Station, United States of America) and accounted for the multistage survey design and sampling weights. In the single-year analyses from 2004 to 2022, due to small samples in some districts (Table 1), we pooled survey results over 6 years (2004–2009) for Hib, followed by 4-year periods (2010–2013, 2014–2017) and a 5-year period (2018–2022) for the three vaccines. Proportions were calculated accounting for the complex sampling design that included weighting and clustering.

**Table 1:** Characteristics of children in annual household demographic and health surveys, Peru, 2004–2022

Characteristic	Year										
	2004–2009 (*n* = 5 257)			2010–2013 (*n* = 7 392)			2014–2017 (*n* = 15 586)			2018–2022 (*n* = 20 788)	
	No.^a^	% (95% CI)		No.^a^	% (95% CI)		No.^a^	% (95% CI)		No.^a^	% (95% CI)
**Vaccine coverage**											
*Hib*	2 869	46.2 (44.1–48.2)		5 386	73.3 (71.9–74.7)		11 817	76.2 (75.1–77.1)		15 592	74.9 (73.9–75.6)
Rotavirus	NA	NA		3 602	47.2 (45.7–48.8)		11 210	72.7 (71.6–73.8)		15 067	72.1 (71.3–73.0)
Pneumococcal conjugate vaccine	NA	NA		3 610	49.2 (47.7–50.8)		11 218	72.2 (71.1–73.3)		14 921	71.7 (70.8–72.5)
***Juntos* enrolment**	NA	NA		1 110^b^	12.3 (11.4–13.3)		2 325	12.8 (12.1–13.6)		2 307^c^	13.5 (12.8–14.1)
**Sex of the child**											
Female	2 617	50.7 (48.7–52.7)		3 721	48.6 (47.0–50.1)		7 627	49.4 (48.3–50.5)		10 561	48.9 (48.0–49.8)
Male	2 640	49.3 (47.3–52.3)		3 671	51.4 (49.9–53.0)		7 959	50.6 (49.5–51.7)		10 227	51.1 (50.2–52.0)
**Wealth quintile**											
1 (poorest)	1 266	19.7 (18.0–21.6)		2 317	24.4 (23.2–25.6)		4 028	21.8 (20.8–22.7)		5 944	23.5 (23.0–24.2)
2	1 443	24.2 (22.4–26.0)		1 950	23.2 (21.9–24.6)		4 163	22.6 (21.6–23.6)		5 633	24.0 (23.2–24.7)
3	1 223	21.3 (19.5–23.3)		1 527	22.6 (21.2–24.1)		3 281	22.0 (21.0–23.0)		4 095	20.3 (19.6–21.1)
4	797	19.4 (17.6–21.4)		999	16.6 (15.3–18.0)		2 440	18.2 (17.2–19.2)		3 009	17.4 (16.7–18.2)
5 (wealthiest)	528	15.3 (13.6–17.2)		599	13.2 (11.9–14.5)		1 674	15.5 (14.4–16.6)		2 107	14.7 (14.0–15.4)
**Area of residence**											
Urban	2 792	58.3 (56.4–60.0)		4 148	65.8 (64.7–66.9)		10 831	73.6 (72.7–74.5)		14 626	75.7 (75.2–76.2)
Rural	2 465	41.7 (40.0–43.5)		3 244	34.2 (33.1–35.3)		4 755	26.4 (25.5–27.3)		6 162	24.3 (23.8–24.9)
**Region**											
Amazonas	230	1.8 (1.5–2.1)		341	1.7 (1.6–1.9)		617	1.6 (1.4–1.8)		846	1.6 (1.5–1.7)
Ancash	207	4.2 (3.6–4.9)		321	4.4 (3.9–4.8)		513	3.4 (3.0–3.9)		723	3.5 (3.3–3.7)
Apurímac	235	2.1 (1.8–2.4)		278	1.9 (1.7–2.1)		517	1.6 (1.4–1.8)		708	1.4 (1.3–1.5)
Arequipa	174	3.4 (2.9–4.0)		229	3.7 (3.4–4.1)		555	4.0 (3.6–4.5)		707	4.0 (3.8–4.2)
Ayacucho	246	2.6 (2.2–2.9)		327	3.1 (2.8–3.4)		577	1.8 (1.6–2.0)		825	1.9 (1.8–2.0)
Cajamarca	220	6.6 (5.8–7.6)		319	5.8 (5.2–6.4)		493	5.1 (4.5–5.7)		697	4.6 (4.4–4.9)
Callao	36	2.7 (2.2–3.3)		68	3.0 (2.5–3.6)		621	3.4 (3.1–3.8)		807	3.4 (3.2–3.6)
Cusco	178	5.1 (4.3–6.0)		259	4.4 (3.9–4.9)		492	3.7 (3.3–4.2)		599	3.4 (3.2–3.7)
Huancavelica	220	2.4 (2.2–2.8)		246	1.8 (1.6–2.1)		488	1.4 (1.2–1.6)		654	1.2 (1.1–1.3)
Huánuco	191	3.4 (2.9–4.0)		263	2.7 (2.5–3.0)		629	2.6 (1.3–3.0)		821	2.7 (2.6–2.9)
Ica	190	2.6 (2.3–3.0)		300	3.0 (2.7–3.3)		645	3.0 (2.8–3.3)		834	3.2 (3.0–3.4)
Junín	178	4.8 (4.2–5.6)		260	4.2 (3.8–4.7)		571	3.6 (3.2–4.0)		747	4.3 (4.1–4.6)
La Libertad	200	6.1 (5.3–7.1)		296	5.7 (5.1–6.2)		624	6.8 (6.1–7.7)		802	6.9 (6.5–7.2)
Lambayeque	176	4.2 (3.3–5.3)		249	3.8 (3.3–4.3)		627	4.0 (3.5–4.5)		821	4.4 (4.2–4.7)
Lima	324	22.0 (20.4–23.6)		541	25.1 (23.9–26.4)		1 750	29.3 (28.8–30.8)		2 601	29.3 (28.6–30.0)
Loreto	318	5.3 (4.7–5.9)		456	5.3 (4.7–5.8)		736	5.3 (4.8–6.0)		852	4.5 (4.3–4.7)
Madre De Dios	297	0.4 (0.4–0.5)		355	3.8 (3.3–4.3)		597	0.6 (0.5–0.7)		757	0.5 (0.5–0.6)
Moquegua	161	0.6 (0.5– 0.6)		185	0.5 (0.4–0.5)		466	0.5 (0.4–0.5)		670	0.5 (0.5–0.6)
Pasco	237	1.1 (0.9–1.4)		296	0.9 (0.9–1.1)		551	0.9 (0.8–1.0)		657	0.8 (0.7–0.8)
Piura	224	7.3 (6.3–8.5)		372	6.9 (6.3–7.6)		667	7.1 (6.4–7.9)		857	7.4 (7.0–7.8)
Puno	203	5.2 (4.6–6.0)		280	4.3 (3.9–4.8)		432	3.4 (3.0–3.8)		546	2.9 (2.7–3.1)
San Martín	212	2.6 (2.2–3.1)		338	3.4 (3.1–3.8)		588	3.0 (2.6–3.3)		772	3.0 (2.9–3.2)
Tacna	126	0.9 (0.7–1.0)		173	0.9 (0.7–1.0)		509	0.9 (0.8–1.0)		707	1.0 (0.9–1.0)
Tumbes	197	0.8 (0.7–0.9)		292	0.9 (0.8–1.1)		630	0.9 (0.8–1.0)		852	0.9 (0.9–1.0)
Ucayali	277	1.8 (1.6–2.1)		348	1.9 (1.7–2.1)		691	2.2 (2.0–2.4)		926	2.4 (2.2–2.5)

CI: confidence interval; Hib: *Haemophilus influenzae* type b; NA: no data available.

^a^ Unweighted sample size.

^b^ 32 values missing.

^c^ Information on *Juntos* enrolment was not available for 2022.

Note: *Juntos* is conditional cash transfers programme, launched in 2005.^16^^,^^17^ Quintiles 1 and 2 represent 40% of the poorest population.

In the ecological analyses, we plotted vaccine coverage against poverty and *Juntos* enrolment in each region. We used linear regression analysis to estimate intercept and slope parameters and calculated Pearson correlation coefficients; we used appropriate transformations when nonlinearity was detected.^23^

For individual-level analyses, we pooled all children in each interval and investigated inequalities according to wealth quintiles, urban–rural residence, child sex and enrolment in *Juntos*. We calculated the slope index of inequality to quantify the absolute difference in vaccine coverage between children at the richest and poorest ends of the wealth distribution. We compared the slope index of inequality using logistic regression analysis of coverage on household wealth based on the whole distribution of children. The index has positive values for pro-rich (when coverage favours richer children) coverage patterns, and negative values for pro-poor (when coverage favours poor children) patterns.^24^

Individual-level analyses included fixed effects for the calendar year and region, and we used sampling weights to reproduce the national population. When pooling results over several years, the existing sample weights also accounted for changes in the national child population. 

All analyses were based on publicly available anonymized databases. The health surveys received ethical clearance from the National Institute of Statistics and Computing and the Peruvian health ministry. 

## Results

Our analyses included 19 annual surveys conducted from 2004 to 2022 with a total of 49 023 children aged 18–29 months. Table 1 shows the number of children analysed in each region and their sociodemographic characteristics, stratified by period. 

### Ecological analyses

#### Hib (pentavalent) vaccine

According to home records, national Hib vaccine coverage was estimated to increase from 27.8% in 2004 to 56.8% in 2009.

**Fig. 1:**
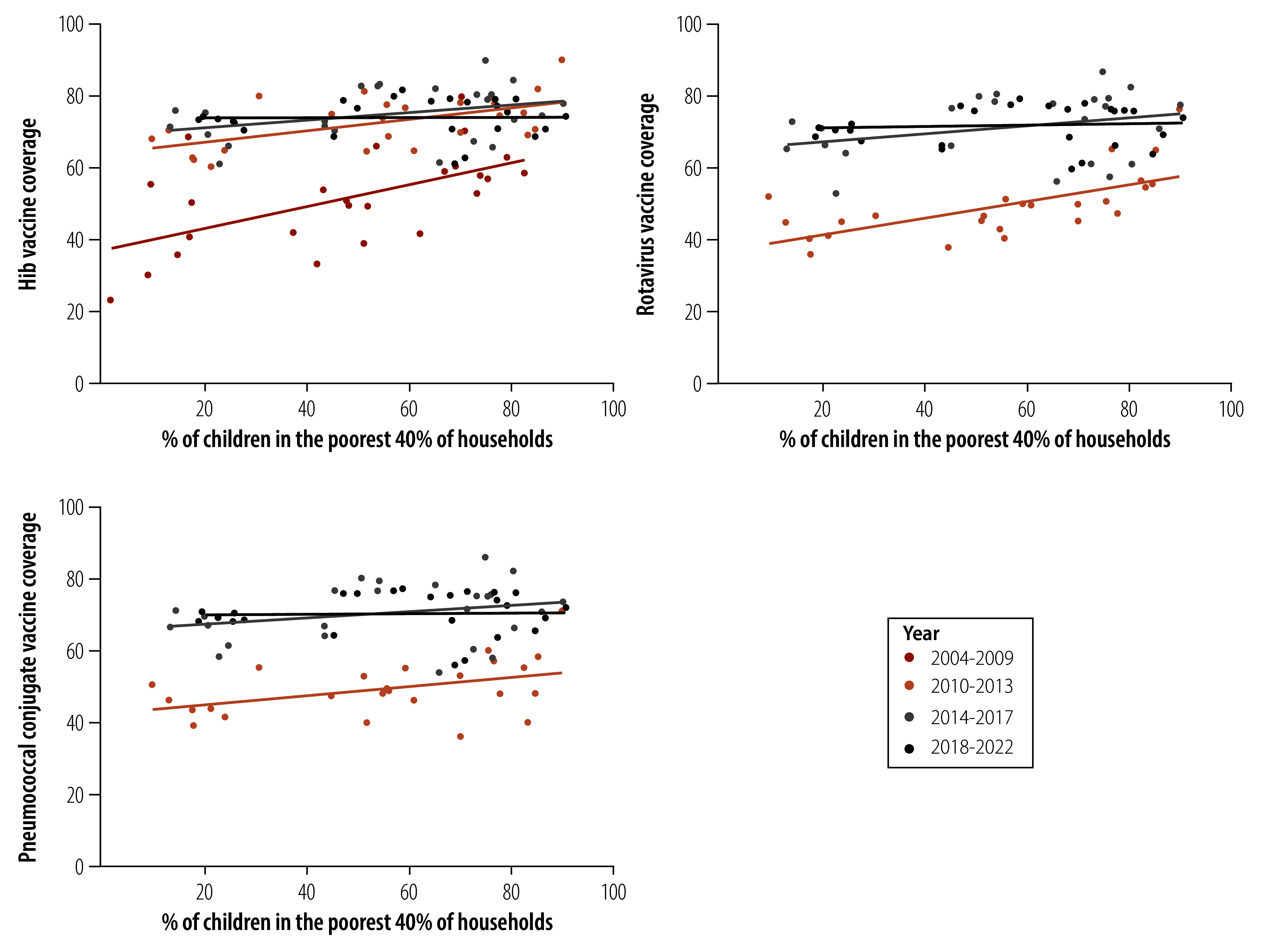
Vaccine coverage of the three vaccines by prevalence of poverty for the 25 regions, Peru, 2004–2022

The relationships between poverty and vaccine coverage are shown in Fig. 1. Each dot represents a region in each period. Steeper line slopes indicate higher coverage in poorer regions, while horizontal slopes suggest similar coverage across all regions (online repository).^21^

Hib: *Haemophilus influenzae* type b.

Note: The prevalence of poverty was calculated as the percentage of children in the bottom 40% of the national wealth index.

The positive slope for the earliest time period (2004–2009) confirms that the poorest regions were prioritized then. However, coverage increased after 2009 and was no longer associated with poverty. The regression coefficients for the different time periods were: 0.31 (95% confidence interval, CI: 0.12 to 0.49) in 2004–2009; 0.16 (95% CI: 0.06 to 0.27) in 2010–2013; 0.11 (95% CI: –0.01 to 0.23) in 2014–2017; and 0.01 (95% CI: −0.09 to 0.10) in 2018–2022.

#### Rotavirus vaccine

By 2010, 16.6% of all children were estimated to have been vaccinated with the rotavirus vaccine. Similar to the Hib vaccine, the initial association with poverty decreased over time (Fig. 1). The regression coefficients were: 0.24 (95% CI: 0.12 to 0.35) in 2010–2013; 0.11 (95% CI: −0.04 to 0.27) in 2014–2017; and 0.02 (95% CI: −0.09 to 0.12) in 2018–2022.

#### Pneumococcal vaccine

By 2010, the national pneumococcal conjugate vaccine coverage was estimated at 11.9%. Fig. 1 shows that also for this vaccine, the direct association of vaccine coverage with poverty in 2010–2013 disappeared over time. The regression coefficients were: 0.13 (95% CI: 0.01 to 0.25) in 2010–2013; 0.09 (95% CI: −0.05 to 0.23) in 2014–2017; and 0.01 (95% CI: −0.10 to 0.12) in 2018–22.

#### Juntos enrolment

Estimates at the national level, showed *Juntos* enrolment increased from 14.3% in 2008 to 18.7% in 2021. Enrolment was associated with poverty (online repository),^21^ but the association was nonlinear, with very high enrolment rates in the poorest regions. The associations between vaccine coverage and *Juntos* enrolment by period (online repository)^21^ tended to be slightly stronger than the associations with poverty, but as was the case for poverty, correlations declined over time and were no longer significant in the most recent period.

### Individual analyses

Individual-level analyses included all children in the national samples. The estimated percentage of children in the poorest 40% of all households ranged from 15.2% in the five richest to 79.1% in the five poorest regions in 2014–2017. We divided the 25 regions into quintiles according to poverty prevalence and found heterogeneity between the regions (online repository).^21^

**Fig. 2:**
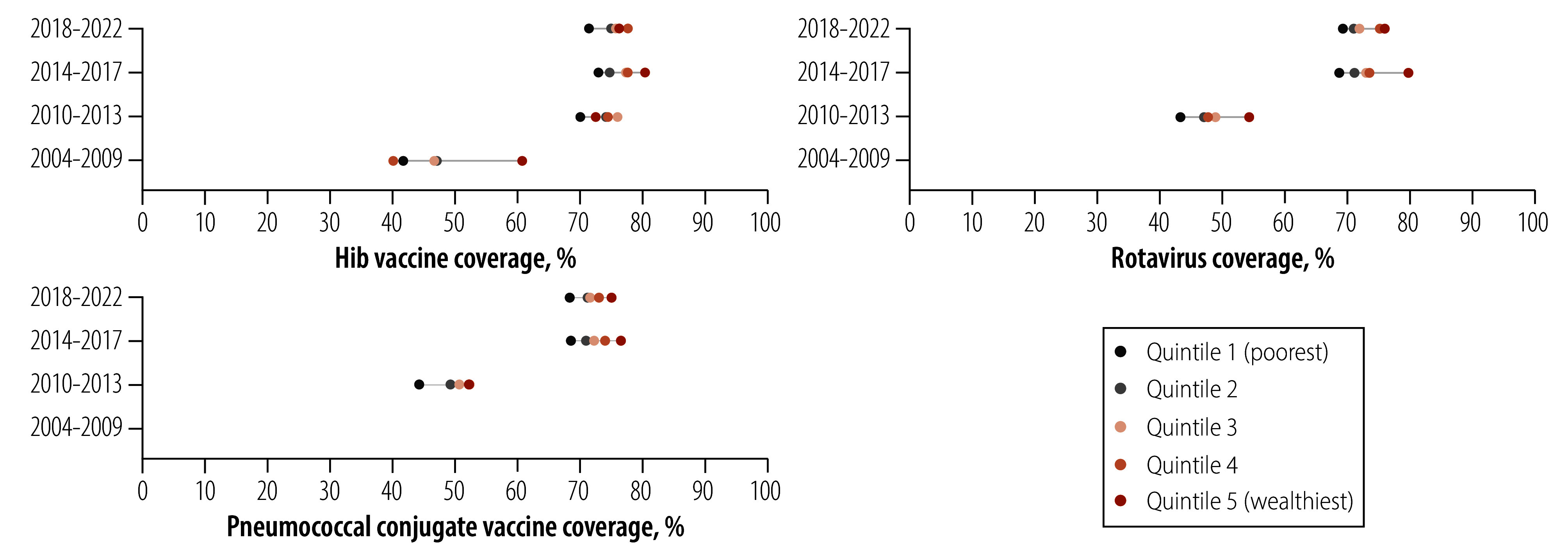
Vaccine coverage of the three vaccines by time period and wealth quintiles, Peru, 2004–2022

Fig. 2 shows that children from wealthier households tended to have higher Hib vaccine coverage in the four periods under study, contrasting with the ecological results for the early periods. The slope index of inequality values were: 13.3 percentage points (95% CI: 4.1 to 22.4) in 2004–2009; 4.8 percentage points (95% CI: −2.2 to 11.8) in 2010–2013; 8.7 percentage points (95% CI: 4.5 to 12.9) in 2014–2017; and 7.1 percentage points (95% CI: 3.4 to 10.8) in 2018–2022, indicating significantly positive associations between coverage and wealth, except for the 2010–2013 period.

Hib: *Haemophilus influenzae* type b.

Note: Individual-level analysis included all children in the national samples.

Rotavirus coverage was positively associated with wealth in the three periods, with slope index of inequality of: 8.9 percentage points (95% CI: 2.7 to 15.0) in 2010–2013; 11.6 percentage points (95% CI: 7.4 to 15.9) in 2014–2017; and 8.6 percentage points (95% CI: 5.1 to 12.1) in 2018–2022.

For pneumococcal conjugate vaccine coverage, the slope index of inequality was: 5.4 percentage points (95% CI: 1.6 to 9.3) in 2010–2013; 9.7 percentage points (95% CI: 5.1 to 14.4) in 2014–2017; and 7.4 percentage points (95% CI: 3.8 to 11.0) in 2018–2022 (Fig. 2).

To investigate the pro-rich patterns further, we selected the five regions with the highest vaccine coverage levels in the early roll-out period for each vaccine (online repository).^21^ Even within the first period, we found no statistical evidence that poverty was positively associated with vaccine coverage in these early roll-out regions. We also compared coverage time trends in the five priority regions and the rest of the country (online repository).^21^ Coverage remained higher in the targeted regions until the coronavirus disease 2019 (COVID-19) pandemic for rotavirus and pneumococcal conjugate vaccine, after which no differences were found. For the Hib vaccine, the gap closed after the first 3 years of implementation.

We used three additional stratifications in the individual-level analyses. Children whose families were enrolled in *Juntos* showed consistently higher coverage than the rest of the population in all periods for all vaccines (all *P* ≤ 0.003; online repository).^21^ Urban children showed significantly higher coverage with pneumococcal conjugate vaccine and rotavirus vaccines up to 2013, after which rural children caught up with them(online repository).^21^ For the Hib vaccine, coverage was similar in urban and rural children. As regards sex differences, we only observed significant differences from 2014 to 2017, when coverage was 2.5 percentage points higher in boys than girls for the Hib vaccine and 2.4 percentage points higher for the pneumococcal conjugate vaccine. We found no sex differences for rotavirus vaccine coverage (online repository).^21^

## Discussion

Health-care systems consistently favour well-off people, offering them more and better services despite their lower health needs than those of poor people.^8^^,^^9^^,^^25^^,^^26^ The Peruvian government initiated a new strategy for vaccine delivery which aimed to reduce child health inequalities by targeting the most vulnerable subpopulations for delivery of new vaccines, and requiring specific coverage targets to be reached for these groups before scaling up national coverage.^9^^–^^11^ Our study assessed the impact of this strategy. The ecological analyses showed strong positive correlations between vaccine coverage and poverty levels in the first few years after introduction. Subsequently, as coverage surpassed 70% in the poorest regions, the roll-out was extended to wealthier regions and these correlations disappeared over time. In contrast with the ecological findings, individual-level analyses showed that children from wealthier families were more likely to be vaccinated than children from poor families in all periods. From 2018 to 2022, coverage in the wealthiest quintile was about 7–8 percentage points higher than in the poorest quintile. Only minor differences were seen by sex of the child, which is consistent with findings from most low- and middle-income countries.^3^

Our findings indicate that although regional disparities were initially reduced, impoverished families may continue to encounter challenges, such as limited access to services, lack of motivation, the necessity to work and other barriers. Nevertheless, the rich–poor vaccine coverage gap in Peru is narrow compared to most low- and middle-income countries, likely due to the innovative approach to vaccine roll-out.^3^

We also studied the potential role of the *Juntos* cash transfer programme. Up to 2021, one of the programme conditions required that young children attend government health services every 1–2 months. Immunizations were only added as a formal condition in 2022,^17^ but the earlier requirement of regular health checks should have provided ample opportunities for vaccination. In addition, due to the targeting criteria,^22^ *Juntos* enrolment rates may have been a better indicator of extreme poverty than the prevalence of households classified as the poorest 40%. Unlike what was observed for poverty in the individual-level analyses, children enrolled in *Juntos* showed higher coverage than the remaining children in all periods for all vaccines. This result suggests that household-level targeting for social benefits contributes to higher coverage, even though not all children from the poorest families were reached.

Although targeted immunization strategies have been implemented in some countries, our study is among the few impact evaluations to have been published. In the Democratic Republic of the Congo, targeting low-coverage districts led to increased immunization rates. However, the study did not report on immunization trends in other parts of the country or on individual-level inequalities.^27^ In India, prioritizing vulnerable states for vaccine scale-up reduced individual-level inequalities in zero-dose status and full immunization coverage.^28^^,^^29^ An earlier Peruvian study on missed vaccination opportunities from 2010 to 2020 showed substantial heterogeneity in the magnitude of individual-level inequalities between different regions. Yet, no clear trends in inequalities over time were observed.^30^ Unlike our study, these analyses were not focused on newly introduced vaccines and none, except for the study in the Democratic Republic of the Congo, attempted to evaluate the impact of targeted implementation strategies.

In introducing this new strategy, the Peruvian government took advantage of the revolving funds initiative coordinated by PAHO. By pooling vaccine orders and negotiating with suppliers, the fund obtained sufficient vaccines at affordable prices for all Member States of PAHO.^31^ Since 2007, the Peruvian government has fully financed its vaccine purchases.^12^ The sustainability of the Peruvian immunization programme is framed within a results-based budgeting approach, through which regional governments and health workers receive financial incentives to reach targets in maternal and child health services.^32^^–^^35^

Monitoring has also played a crucial role. Our analyses were made possible by the availability of annual household surveys since 2004, which makes Peru a global example of the importance of collecting population-based data to guide health policies. The national government also monitors the impact of multisectoral programmes through national surveys of budgetary programmes which have been conducted since 2010.^36^

Our analyses have some limitations. The small number of children sampled from each year in some of the least populated regions affected the precision of the annual estimates. This limitation was addressed by aggregating results over 4 to 6 years. Poverty was assessed using asset indices that may underestimate the wealth of rural households. Still, a scaling procedure was used to improve comparability with urban families.^22^ Vaccine coverage levels were derived solely from home-based records, as information on reported doses was unavailable for the pneumococcal conjugate and rotavirus vaccines. Based on data for the Hib vaccine, levels reported here may be lower than true coverage levels. Nevertheless, this restriction should not affect our results on trends and associations. In addition, because secondary data from health information systems do not contain information on wealth, residence or *Juntos* enrolment, individual-level analyses were necessarily limited to survey data, which provide such information. 

Based on our findings, policy-makers and health managers may wish to consider the following actions. First, identify the most vulnerable regions in their country based on poverty and child mortality rates and prioritize these geographic regions for vaccine supply, expansion of the cold chain, and staff deployment and training. Second, complement geographic targeting with a household-level strategy, for example, by using listings of vulnerable households enrolled in social protection programmes. Government social workers and community health agents will be needed to deliver vaccines at home or to bring these children to health facilities. Third, ensure regular data collection to monitor coverage and inequalities by reinforcing existing health information systems to allow stratified analyses, if possible, complemented by purposefully designed household surveys. Fourth, ensure sustainability by procuring long-term funding to maintain early gains over time. Lastly, work with other governmental and nongovernmental agents to develop multisectoral policies for poverty reduction.

To conclude, Peru has been affected by frequent changes at the top managerial level of the health ministry. Nevertheless, the country’s health systems are resilient, and have resulted in improved child health, lower mortality and undernutrition, and high and equitable maternal and child health services coverage.^10^^,^^37^ In Peru, targeting vaccination at the regional level was highly effective in the first years after introducing new vaccines and, over time, led to equitable and high coverage throughout the country. Nevertheless, geographic targeting failed to eliminate the gaps in coverage between children from wealthy households and those from poor households. Although these gaps are relatively small they remain a barrier to universal coverage.
